# Simulation and Optimization of Electromagnetic Absorption of Polycarbonate/CNT Composites Using Machine Learning

**DOI:** 10.3390/mi11080778

**Published:** 2020-08-15

**Authors:** Lakhdar Sidi Salah, Mohamed Chouai, Yann Danlée, Isabelle Huynen, Nassira Ouslimani

**Affiliations:** 1 Laboratory of Coatings, Materials and Environment, M’Hamed Bougara University, Boumerdes 35000, Algeria; l.sidisalah@univ-boumerdes.dz (L.S.S.); ouslimaniboumerdes@yahoo.fr (N.O.); 2 Signals and Systems Laboratory, Department of Electrical Engineering, Mostaganem University, Site 1 Route Belahcel, Mostaganem 27000, Algeria; 3 ICTEAM Institute, Université Catholique de Louvain, 1348 Louvain-la-Neuve, Belgium; yann.danlee@uclouvain.be (Y.D.); isabelle.huynen@uclouvain.be (I.H.)

**Keywords:** artificial intelligence (AI), multilayer perception (MLP), electromagnetic interference (EMI) shielding, nanocomposite, absorption index, carbon nanotubes (CNTs), polycarbonate (PC), Rozanov formalism

## Abstract

Electronic devices that transmit, distribute, or utilize electrical energy create electromagnetic interference (EMI) that can lead to malfunctioning and degradation of electronic devices. EMI shielding materials block the unwanted electromagnetic waves from reaching the target material. EMI issues can be solved by using a new family of building blocks constituted of polymer and nanofillers. The electromagnetic absorption index of this material is calculated by measuring the “S-parameters”. In this article, we investigated the use of artificial intelligence (AI) in the EMI shielding field by developing a new system based on a multilayer perceptron neural network designed to predict the electromagnetic absorption of polycarbonate-carbon nanotubes composites films. The proposed system included 15 different multilayer perception (MLP) networks; each network was specialized to predict the absorption value of a specific category sample. The selection of appropriate networks was done automatically, using an independent block. Optimization of the hyper-parameters using hold-out validation was required to ensure the best results. To evaluate the performance of our system, we calculated the similarity error, precision accuracy, and calculation time. The results obtained over our database showed clearly that the system provided a very good result with an average accuracy of 99.7997%, with an overall average calculation time of 0.01295 s. The composite based on polycarbonate−5 wt.% carbon nanotube was found to be the ultimate absorber over microwave range according to Rozanov formalism.

## 1. Introduction

Since 2019, artificial intelligence (AI) is in the spotlight. This technology is developing at high speed, and its use cases are increasing in all sectors. According to experts, AI is set to overturn all aspects of our society in the years to come [1].

An immense variety of static phenomena can be characterized by a deterministic relationship between causes and effects; neural networks are good candidates for modeling such relationships from experimental observations, given that there are sufficiently numerous and representative data [2].

AI algorithms and machine learning are making headlines in several areas, such as commerce, medicine, education, autonomous driving, entertainment, etc. AI does not leave aside the field of scientific research, where it imposes itself as an essential tool for scientific discovery. The new AI method has been developed by an interdisciplinary team of chemists, physicists, and computer scientists from the University of Warwick, the Technical University of Berlin, and the University of Luxembourg [3]. According to this article [3], artificial intelligence has become, in chemistry and applied physics, an essential tool for predicting the results of experiments or simulations.

Artificial intelligence and machine learning algorithms continue to catalyze discoveries in the field of scientific research. A recent method developed by researchers has shown that AI can be used to predict the quantum states of molecules, called wave functions, which determine all the properties of molecules. According to the researchers behind the discovery, their algorithm could also significantly speed up future simulation efforts in the design of drug molecules or new materials [4]. Algorithms are recently developed for the optimization of specific applications like conductive composites for electrochemistry [5].

Among the methods of artificial intelligence is the multilayer perceptron (MLP), which is a computer system inspired by the functioning of the human brain to learn [6]. In principle, MLP is nothing more than a way of constructing parametric models, i.e., a model where the decision function is explicit. Unlike other parametric algorithms, such as linear regression, MLP allows to easily build very complex and non-linear models [7].

**Figure 1:**
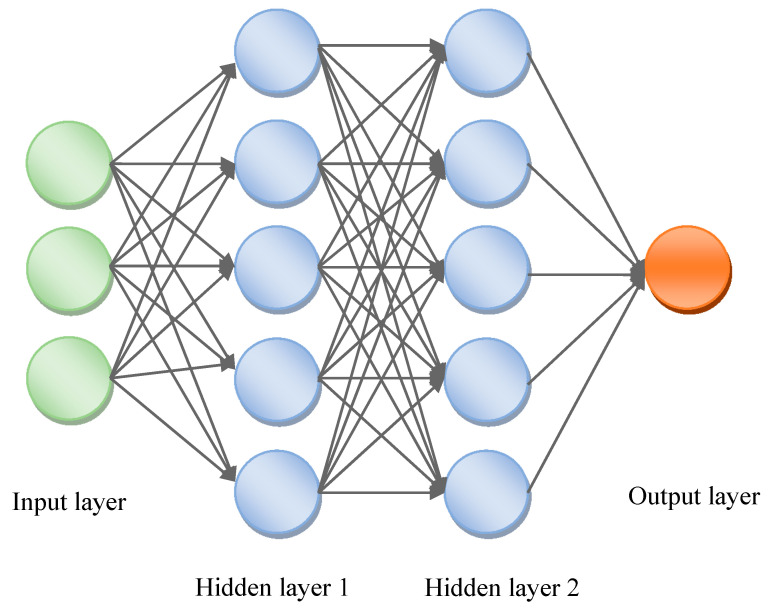
Block diagram of a multilayer perceptron. Adapted from Al-Naymat, G., et al. Classification of VoIP and non-VoIP traffic using machine learning approaches [8].

The MLP works as follows: typically, a neural network relies on a large number of processors operating in parallel and organized into three stages. Input and output layers shown in Figure 1 are connected by hidden layers that perform non-linear transformations of the inputs entered into the system network. The first layer receives raw information input, like the human optic nerves, when processing visual signals. Subsequently, each layer receives information from the previous one. The same process is found in the human body when neurons receive signals from the neurons close to the optic nerve. The last layer produces the results of the system. Figure 1 shows the MLP scheme.

Unlike other types of algorithms, neural networks cannot be directly programmed to perform a task. Like a developing child’s brain, the first assignment is to understand the basic mechanism of their environment to get useful information. Through an algorithm, the artificial neural network allows the computer to learn from new data. The computer with the neural network learns to perform a task by analyzing examples for training. These examples have been labeled beforehand so that the network knows the output corresponding to a specific input set and then fine-tune the network.

Polymer nanocomposites, consisting of nanoparticles dispersed in a polymer matrix, have gained large interest due to the attractive properties of nanostructured fillers, which are embedded in low volume fraction (1–5%) into the polymeric matrix, providing important electrical properties, which allow the insulating matrix to pass from dielectric to semi-conductive class [9]. Multi-walled carbon nanotubes (MWCNTs) are highly used in the fabrication of structural composites with remarkable properties, allowing these composites to be used in many applications, such as electromagnetic induction shielding [10]. The performance of polymer-nanofiller composites to block electromagnetic (EM) radiation is evaluated from measuring a physical electromagnetic absorption index property using S-parameters procedure; these parameters have been extracted from a highly sensitive powerful instrument, namely, a vector network analyzer (VNA). This machine utilizes the concept of measuring the transmitted and reflected waves as a signal passes through a device under test [11]. This measurement method is used on a large scale of recent research; it is considered as a shorthand way to identify the absorption index over large selective frequencies based on S-parameters [12,13].

Based on the multiplicity of electromagnetic absorption index values over a different large of frequencies for various polycarbonate/MWCNTs film composites characterized by a VNA instrument, the implementation of a computing new design is needed. It aims to give the same performances as a VNA analyzer and a prior knowledge of electromagnetic absorption index for researchers who investigate on EMI shielding field of polycarbonate (PC)/carbon nanotube (CNT) microdevices. This method can suppress multiple series of tests, as well as calculus procedure of raw data conversion to physical parameters (e.g., absorption index, shielding effectiveness, permittivity, etc.), and calibration of the machine prior to use, adding to all of these characteristic parameters: time measurement dependency and requirement of a qualified operator having the ability to resolve the problems that may survey during the measurement procedure. The development of a new numerical method consisting of a multilayer perceptron neural network, aiming to predict the electromagnetic absorption index at an identified frequency, is designed to surpass the above issues. This method has been chosen due to the large and different scale of input data as it is presented in Table 1 in materials and data collection section; MLP has been shown to approximate virtually any function to any desired accuracy; however, this is validated only if the number of training data in the series is sufficiently large, which acquire a good understanding for the MLP network to learn the required input-output relationship accurately [14].

This paper is organized as follows: Section 2 sets the problematic and the objective and then shows the motivation and the originality of this work; Section 3 presents the materials and data collection used in this study; Section 4 gives a description of the different phases involved in the proposed method, including the background information, implementation details of the evaluation procedure; Section 5 introduces the Rozanov formalism used for gauging the absorption performance; Section 6 presents the experimental part in which the results are discussed; finally, Section 7 closes the article with conclusions and future work.

## 2. Problematic and Objective

Any electronic device that produces, transports, and consumes electrical energy generates electromagnetic interference (EMI); this latter can cause malfunctioning and device damages, as well as an increase in electromagnetic pollution, affecting human health if no shielding is provided [15]. EMI shielding materials are designed to block harmful electromagnetic radiation from reaching the target material. The shielding efficiency is related to the electrical conductivity of the material [16]. Metals, such as silver and copper, due to their high EMI shielding efficiency, are a natural solution to mitigate EMI issues. However, the high density, the high cost, the processing difficulty, and the corrosion ability limit the use of metals for the next generation of mobile electronic and communication devices [17]. EMI issues can be solved by utilizing a new family of building blocks constituted of conductive nanofillers, such as multi-walled carbon nanotubes (MWCNTs) combined with a polymer, such as a polycarbonate (PC). These polymer-nanofillers composites are particularly adapted to interact with microwaves through their easily controllable conductivity [18]. One of the highest challenges is to minimize the thickness of the absorbing materials to make them compatible with the targeted components, which are often microscopic. EMI shielding is, as well, required for large sensitive equipment and instrument. The manufacturing of the absorber must be feasible at different scales, and the absorber must preferably be conformable to an arbitrary shape. Those conditions force to select appropriate material and optimize its structural organization to reach the objectives [19]. In this work, the polycarbonate/CNT composites are synthesized using the melt blending process, as described in Section 3 [19]. The electromagnetic absorption index is calculated from measured S-parameters according to [20]:

(1) $$A=1-{\left|S_{21}\right|}^{2}-{\left|S_{11}\right|}^{2}$$

In this article, we investigated the use of AI in the EMI shielding field by developing a new system based on a multilayer perceptron (MLP) neural network (NN) method, designed to predict the electromagnetic absorption of PC/CNT composites films reinforced by different weight percentage of carbon nanotubes.

The state-of-the-art shows that machine learning techniques have never been used in the absorption index prediction of polycarbonate/CNT composites. This can be explained by the lack of sufficiently large databases.

The originality of this work is described hereafter. The objective was to obtain by predictions using the AI MLP algorithm the absorption index in an automatic way without having to go through the measurement procedure (VNA analyzer). The idea was to apply a machine learning method to the prediction process, exploiting the data values of PC/CNT composites films’ information of different samples. As we assumed to have a priori knowledge of the absorption index of each input, it was possible to predict the absorption index of a new composite by means of supervised machine learning. Finally, the optimal concentration of carbon nanotubes for maximum EM absorption was determined by a method independent from geometrical aspects (such as thickness) and frequency band. The efficiency of any absorber was determined by Rozanov formalism, which is used as the reference in the domain [21]. The objective was to obtain the thinnest absorber, showing the highest possible absorption over a defined frequency band (the equation sharply favored a wide band of operation).

## 3. Materials and Data Collection

The polymer matrix used for the conductive nanocomposites was Bayer Makrolon^®^ OD2015 polycarbonate. The CNTs were multi-walled NC 7000 nanotubes provided by NanoCyl SA, Belgium. The nanocomposite compounds were melt-blended at 280 °C for 5 min at 150 RPM in a micro 15 DSM micro-compounder. The composite pellets were twice hot-pressed under six different operating conditions adapted to the viscosity of the pellets in a Fontijne press to produce films with various thicknesses, as shown in Table 1. It is important to mention that *PC-xxxCNT* and *T°/press./time* represents the composite name and the pressing conditions (temperature, pressure force, and pressing time, respectively).

The electromagnetic characterization is made by an Anritsu M54644B in waveguide configuration described in Ref. [22]. The calibration was made by LRL/LRM method for each frequency band, and the IF bandwidth was set at 300 Hz. An aluminum plate was used as a PEC (perfect electric conductor) against the port 2 for the Rozanov measurements.

In the current work, 34,362 experimental data points for the electromagnetic absorption index of PC/CNT composite films were derived using the method described in Ref. [1] from the measured S-parameters at multiple ranges of frequencies. Table 1 sums up the observed range of experimental measurements. The EM absorption index, expressed in % from Equation (1), was the final parameter that we wanted to predict based on the corresponding inputs, namely, frequencies, pressing process conditions, effective conductivity, the weight of CNT (wt.%), and the thickness of the composite film.

## 4. The Proposed System

**Figure 2:**
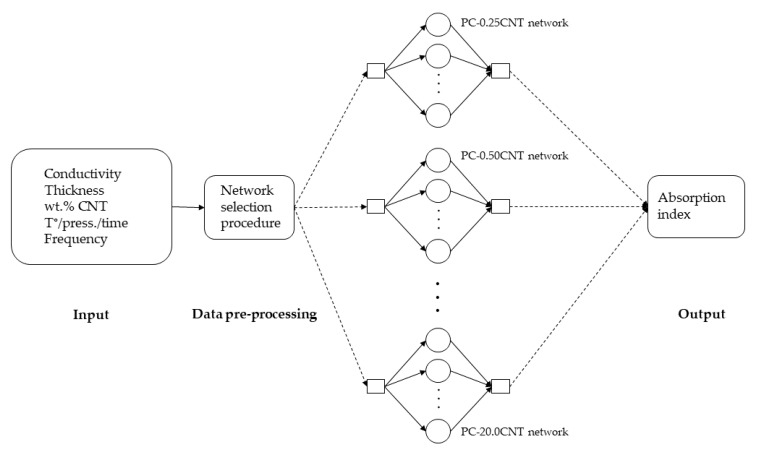
Synoptic diagram describing the steps of the proposed system.

The complete system included 15 different MLP networks; each network was specialized to predict the absorption value for each composite. Figure 2 shows a synoptic diagram describing the steps of the proposed system. The complementary explanations about the architecture of the MLP are given in Section 4.1. The selection of appropriate networks was done automatically, using an independent pre-processing block, used to process the input variable to know which *PC-xxxCNT* film the user has chosen.

Optimization of the hyper-parameters using hold-out [23] validation was required to ensure the best results. The data set was divided into two sets (training and testing). The input sample was the frequency, and the target indicated the corresponding value of the absorption index. Once the datasets were prepared, the search for the optimal hyper-parameter values (number of neurons of MLP) of each network was performed.

### 4.1. MLP Neural Network

The capacity of nonlinear classification/prediction methods seems to be particularly interesting and powerful. However, before implementing such models, it is necessary to ensure the necessity of nonlinear properties of the proposed model. Indeed, if the prediction does not require a particular complexity, the use of linear methods would be more judicious. If the use of nonlinear methods is necessary, the choice between several machine learning methods cannot primarily be decided before an understanding of their applicability in modeling a specific issue, as applied in this article [24].

For our study, beforehand, we reported results of a comparative study of widely used machine learning algorithms applied to predictive polycarbonate-CNT composites absorption index. The machine learning algorithms involved were chosen in terms of their representability and diversity, were extensively evaluated with seven data sets that were taken from real-world applications (we chose to work with Support Vector Machine with three different kernels, Random Forest, Naive Bayes, K-Nearest Neighbors, and Linear Discriminant Analysis). Some interesting findings relating to the data and the quality of the models were obtained in advance; MLP seemed the best for the several machine-learning methods tested in which we obtained the best accuracy with a minimized calculation time.

We chose to work with the MLP network because it gives us the advantage of the following three main points

- Flexibility of neural networks

MLP has the capacity to deal with a wide variety of problems. The result can be a prediction, classification, or data analysis. They make it possible to deal with unstructured problems, where no information is available beforehand [25].

- Yielding of important experimental results

MLP gives good results because, even in very complex fields, they are more efficient than statistics or decision trees. By discovering the relationships between the variables, the implemented system does not force us to question the form of the function to be estimated [26,27,28].

- Relevance of the analyzed data

MLP can work on incomplete or noisy data. This data imperfection can be filled by adding additional neurons to the hidden layer [29].

#### Implementation of the MLP Neural Network

The implementation of multilayer neural networks includes two design parts: first, the objective is to lead toward the best choice of architecture, then a part of numerical calculation carries out the learning of a neural network. The procedure can be divided into four stages:

- **Step 1:** Setting the number of hidden layers

Aside from the input and output layers, the analysis must decide the number of intermediate or hidden layers. Without a hidden layer, the network offers only weak possibilities of adaptation. With one hidden layer, it is capable, with a sufficient number of neurons, of approximating any continuous function. A second hidden layer takes into account any discontinuities.

- **Step 2:** Determining the number of neurons

A larger number of neurons makes possible a better stick to the data presented but decreases the generalization capacity of the network. Consequently, we varied the number of neurons and calculated the mean square error (MSE) to choose the optimal number, leading to the best performance and minimizing the calculation time.

- **Step 3:** Choosing the activation function

In this work, we considered the sigmoid function [30] for the transition from the input layer to the hidden layer, and the transition from the latter to the output layer was linear.

- **Step 4:** Choosing the learning

Supervised learning is applied by providing the network with the inputs and, at the same time, the desired outputs. The network must be trained by adjusting its weights so as to reduce the difference between the desired output and its actual output; this procedure is repeated until a performance criterion is satisfied. The learning algorithm used in our work was that of backpropagation of the error because the latter is the most suited for learning MLP-type neural networks [31].

### 4.2. Evaluation

In general, to evaluate the performance of a machine learning system [32], we divided the sample of data already classified and available into two sets: the training set on which the system learns, and the test set on which we assess its performance. The test set contained the information of the sample; we knew in advance its absorption index at which it should be reached. We were thus able to compare the decisions made by our automatic prediction system, with real data, and then calculate the performance score.

Different measures can be calculated to assess the performance of a system; for our study, we were interested in the similarity error between the actual absorption index and that predicted, thus, the response time of the system. So-called x is the actual value of the absorption index, y is the predicted one, L is the number of tested samples; the similarity error (Err) and the precision accuracy (P) are calculated as follows:

(2) $$\mathrm{Err}=\frac{\sum \left|x-y\right|}{L}$$

(3) P=100−Err%

## 5. Prediction of the Optimal Weight Loading of CNT Using Rozanov Formalism

The previous section investigates the role of machine learning in predicting the EM absorption index of PC/CNT composites over a wide range of frequencies. As can be seen in Table 1, EM absorption is related to film thickness and CNT weight loading. The Rozanov formalism, introduced in Ref. [21], is a useful way to investigate the intrinsic absorption of each composite without artifact due to the thickness. It is important to interpolate this experimentally in order to predict the critical wt.% loading of CNT and the ultimate film thickness for optimal electromagnetic absorption. This section is presented as follows:

1. The measurement of the reflection coefficient of PC/CNT films back coated with a PEC was performed using a vector network analyzer (VNA) and a waveguide configuration.
2. The Rozanov performance was calculated according to:

(4) $$\left|\int_{0}^{\infty }\ln\left|R\left(\lambda \right)\right|d\lambda \right|\leq 2\pi^{2}\overset{N}{\underset{n=1}{\sum }}\mu_{s,n}d_{n}$$

where $\mu_{s,n}$ is the relative static permeability equals to 1, $d_{n}$ is the layer thickness, N is the number of film layers, which was equal to 1 in our study, λ is the wavelength, and R is the reflection coefficient [33].

By introducing the constant parameters ($\mu_{s,n}$ and N) and rearranging, Equation (4) can be written as:

(5) $$d_{\mathrm{Rozanov}}=\frac{\left|\int_{0}^{\infty }\ln\left|R\left(\lambda \right)\right|d\lambda \right|}{2\pi^{2}}$$

3. This equation was used to calculate the theoretical thinnest film of PC/CNT composite following the Rozanov formalism.
4. Finally, a graph of the figure of merit (FOM) defined by the ratio

(6) $$\mathrm{FOM}=d_{\mathrm{Rozanov}}/d_{\mathrm{sample}}$$

was drawn versus wt.% CNT in order to evaluate the performance of each composite. The optimal concentration of CNT loading to achieve the Rozanov limit of absorption was extrapolated from the graph using a polynomial function curve.

## 6. Results

Many arrangements were tested to find an MLP architecture that provides a satisfactory performance without high computational effort and minimizing the calculation time.

It was observed that when using more than ten neurons in the hidden layer and more than one hidden layer, there were no significant changes in the performance. Therefore, architectures with more than one hidden layer should be eliminated, as this would only increase the computational effort.

The networks were trained on an Intel core i5-4200m 2.5 GHz CPU with 4 GB of RAM. We stopped the training when there was no longer decrease in the loss.

Table 2 shows the precision of our system applied to the different neural networks, as well as the calculation time of each network (the calculation time of the preprocessing step was 0.00451 s). We can clearly see that the system provided a very good result with an average accuracy of 99.7997%; the overall average calculation time of the system, if we include the preprocessing time, was 0.01295 s. This duration assessed the performance of our method to predict the same results as measurements. Our method was time saving compared to the time-consuming calibration of the VNA analyzer prior to the measurement of S-parameters yielding to the absorption index. The interest of our approach was that the trained optimized NN could be used to predict the performance of any CNT absorber without performing time-consuming validation measurements. Coupled with the Rozanov formalism (4–6), it could be used for the optimization of the absorber prior to its fabrication. Based on the above discussion, we were convinced of the superiority of our method compared to trial-and-error successive fabrications. Meanwhile, a more detailed evaluation in real-time laboratory conditions could be useful to validate further our approach.

**Figure 3:**
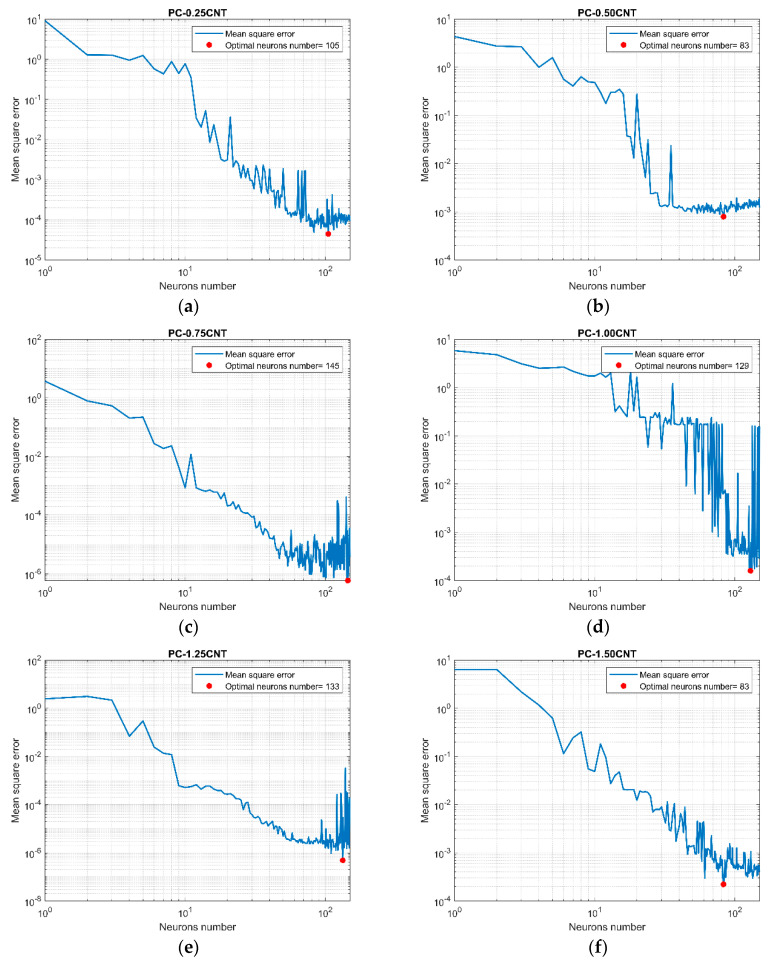

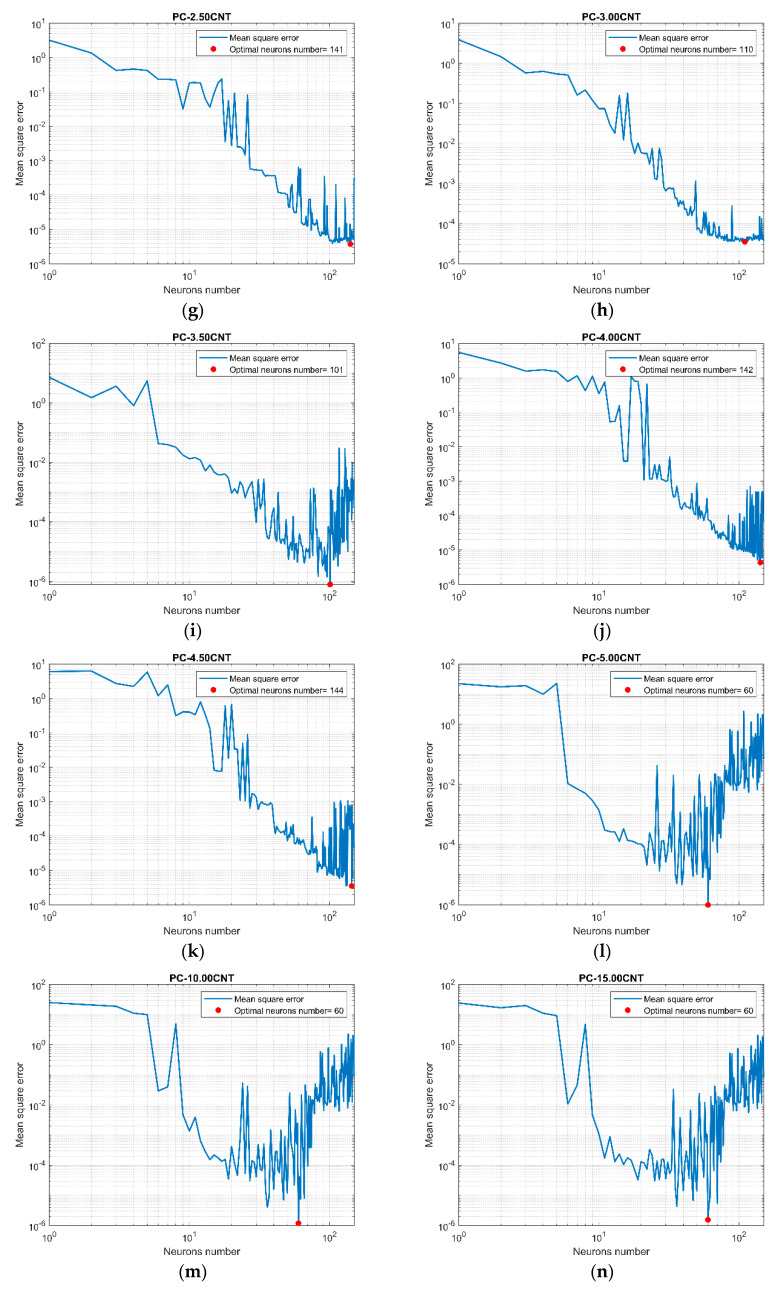

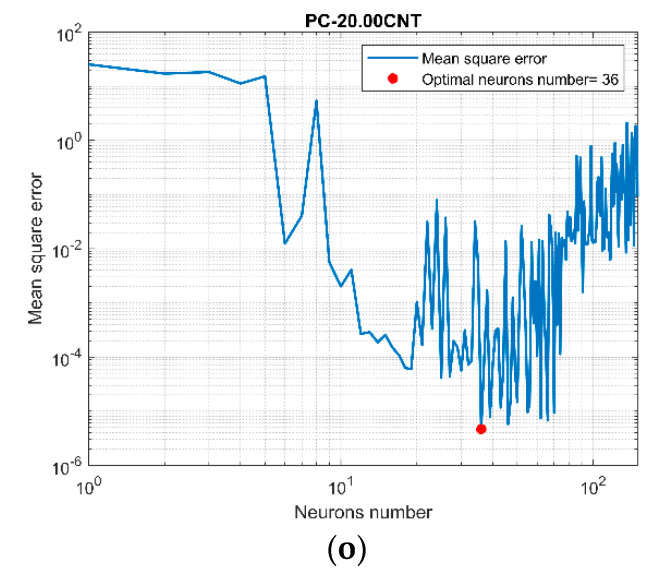
Logarithmic scale plot showing the hyper-parameter optimization process via the hold-out validation method. Figures show the optimal values (red points) of the number of neurons of multilayer perceptron (MLP) from the lowest carbon nanotube (CNT) load (**a**) up to the highest load (**o**).

Arbitrary use of hyper-parameters in different machine learning algorithms leads to overfitting and underfitting problems, the main cause of the poor performance of predictive models. Figure 3 shows that overfitting occurred when the number of nodes of the MLP networks exceeded the optimal one, and underfitting occurred for a number below this value.

**Figure 4:**
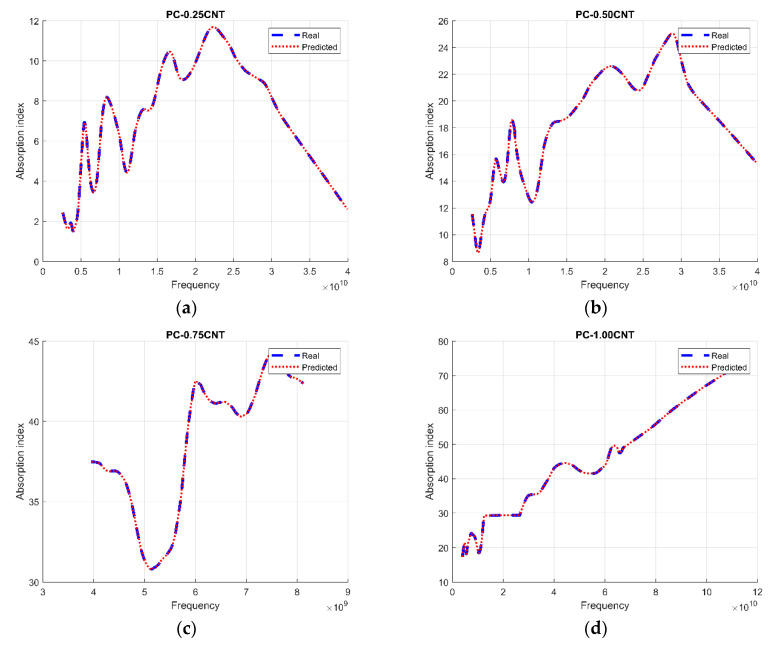

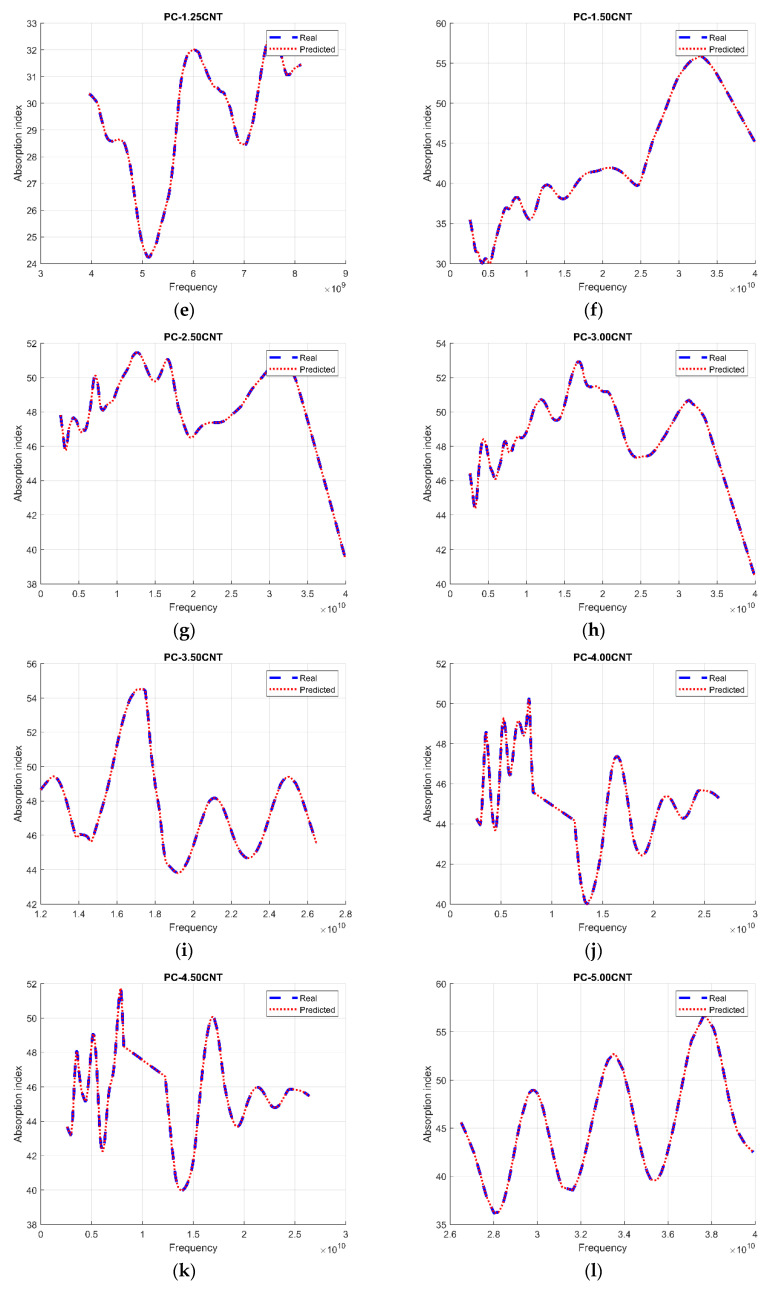

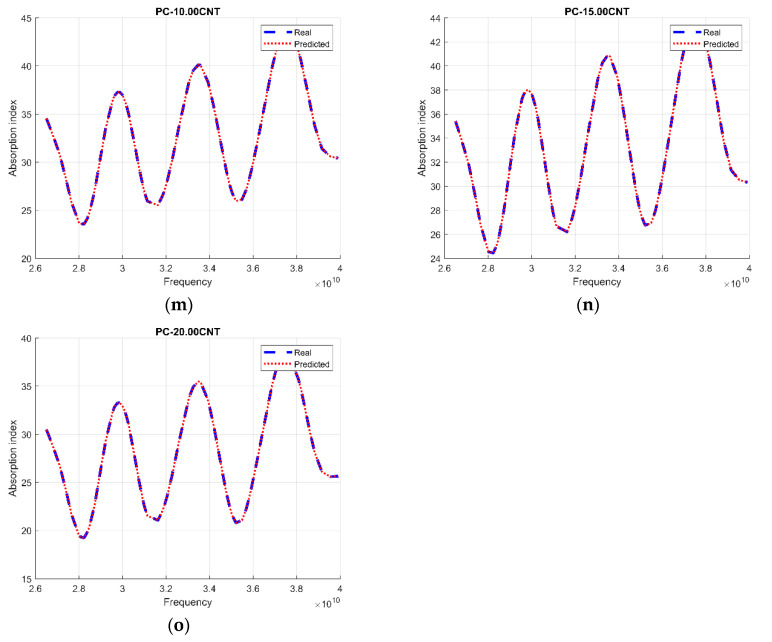
System evaluation on the test set, the blue and red lines, respectively, show the actual and predicted absorption of the measured sample MLP from the lowest CNT load (**a**) up to the highest load (**o**).

Figure 4 shows the process response on the test set; a mean similarity error of ±0.002003 was found for our system. The present study demonstrated that the nonlinear system based on an MLP model was very useful to approximate the absorption index. This technique proved to be successful when the operating conditions were the same that were employed for training purposes.

**Figure 5:**
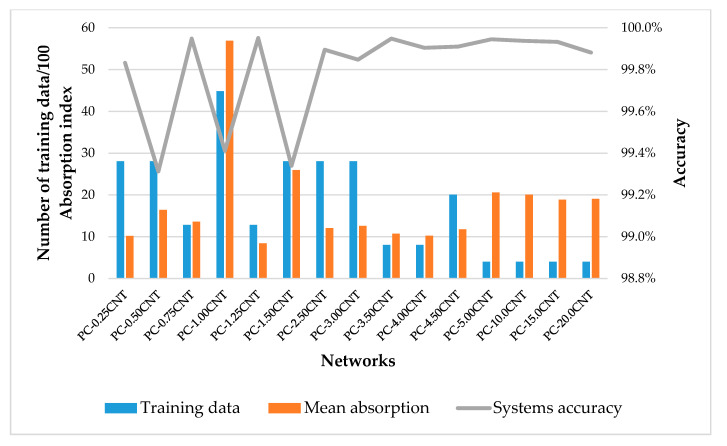
Relationship between training data, the mean absorption index from Table 1, and systems accuracy.

From the Figure 5, we reported that there was an inverse relationship between the range of absorption indices and the accuracy of the systems, i.e., while the interval of the training data of the absorption index decreased, the performance of the system increased, and this relationship was influenced by database’s richness. The PC-3.50CNT network, despite these only 802 training points in its database, had the best performance with 99.95%; this was due to the small interval of its absorption index. In addition, the PC-1.00CNT network had a lower performance than the PC-3.50CNT network (99.41%) in spite of its database richness; this was because of its wide index absorption range. This behavior was the consequence of the impact of the size of the data set and its diversity on the skills and performance estimates of the machine learning model [34,35].

**Figure 6:**
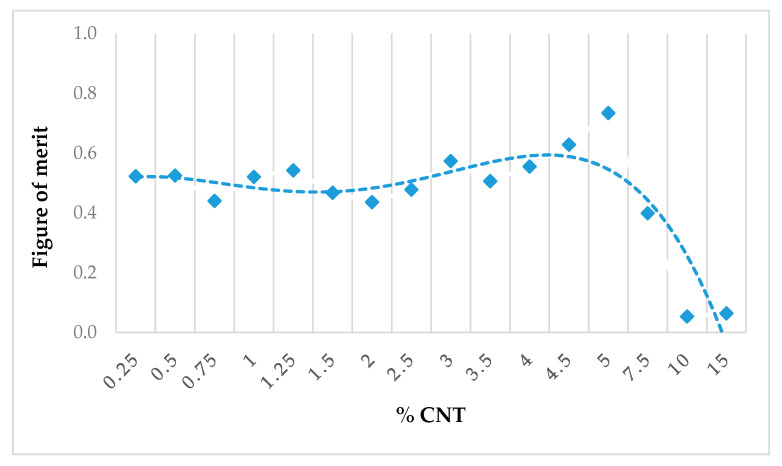
FOM (figure of merit) versus wt.% CNT. Symbols: measurement, dashed line: polynomial fit.

Additionally, the FOM based on Rozanov formalism described in Section 5 is displayed in Figure 6 for each studied composite. The bullet corresponded to the measurement, and the dashed line to its polynomial fit; the best performance was obtained by the film of 5 wt.% CNT with a figure of merit reaching 0.73 (knowing that the theoretical maximal value should be equal to 1). It proved that the most efficient rate of CNT was 5 wt.% for maximal absorption over microwave range with a homogeneous PC/CNT composite obtained by the blend extrusion process. Let’s note that the effective absorption index can be improved by increasing thickness (at the expense of the FOM) or by implementing a specific structure (e.g., split-ring resonators, metamaterial, etc. [22,36]).

## 7. Conclusions

This paper proposed the study of polycarbonate-carbon nanotubes conductive composite films as absorbers of electromagnetic interference at a large band of frequencies. Prediction and simulation of the electromagnetic absorption using AI were the purposes of this study. For this reason, the design of a new system based on an MLP method was considered. A series of evaluation and optimization was realized, aiming to minimize both mean fitting error and calculation time to ±0.002003 and 0.01295 s, respectively. The use of AI models is a new insight into the next studies of PC/CNT composites for EMI shielding applications. The present work showed that the composite PC-5 wt.%CNT reached the best absorption index following the Rozanov formalism with the implementation process by hot extrusion. Future work will be carried out, focusing on the modeling of the degree of percolation and dispersion of the CNT in the polycarbonate matrix using image processing and deep learning. Besides, a deeper real-time validation of our approach could be carried out via the comparison between time-dependent VNA measurements of absorption with its simulation time using our MLP algorithm.

Overall, the main contribution and originality of our work compared to the state-of-the-art are the combination of MLP algorithm and Rozanov formalism, allowing us to gauge and optimize the performance of CNT-based absorbers, prior to their fabrication.

## Figures and Tables

**Table 1:** Representation of the input and output data values of PC/CNT composites films.

Formulation Designation	Freq. Range (GHz)	Av. Conduct. (S/m)	Thick. (µm)	wt.% CNT	Absorption Index (%)	No. of Data	Pressing Conditions (T°/Press./t)
PC-0.25CNT	2–40	1	125	0.25	1.516–11.689	3507	290 °C/7.5T/2.5 min
PC-0.50CNT	2–40	3	135	0.50	8.645–25.066	3507	
PC-0.75CNT	2–40	8	360	0.75	30.804–44.389	1602	250 °C/10T/3 min
PC-1.00CNT	4–115	5	175	1	17.258–74.173	5607	290 °C/7.5T/2.5 min
PC-1.25CNT	2–40	6	530	1.25	24.228–32.661	1602	250 °C/10T/3 min
PC-1.50CNT	2–40	10	130	1.50	30.007–55.943	3507	290 °C 7.5T 2.5 min
PC-2.50CNT	2–40	28	180	2.5	39.397–51.462	3507	
PC-3.00CNT	2–40	45	140	3	40.361–52.931	3507	
PC-3.50CNT	12–26	65	140	3.50	43.805–54.509	1002	
PC-4.00CNT	2–26	85	155	4	40.005–50.244	2505	
PC-4.50CNT	2–26	90	145	4.50	39.964–51.732	2505	
PC-5.00CNT	26–40	80	400	5	36.104–56.713	501	280 °C/10T/2 min
PC-10.0CNT	26–40	110	~160	10	23.470–43.539	501	
PC-15.0CNT	26–40	125	~160	15	24.364–43.226	501	280 °C/10T/3 min
PC-20.0CNT	26–40	130	~160	20	19.184–38.222	501	280 °C/10T/4 min

**Table 2:** The precision of our system applied to different neural networks.

Name	Precision Accuracy	Calculation Time
PC-0.25CNT	99.8325%	0.009921 s
PC-0.50CNT	99.3121%	0.010471 s
PC-0.75CNT	99.9481%	0.007242 s
PC-1.00CNT	99.4115%	0.009182 s
PC-1.25CNT	99.9511%	0.007256 s
PC-1.50CNT	99.3395%	0.008063 s
PC-2.50CNT	99.8953%	0.010935 s
PC-3.00CNT	99.8475%	0.007603 s
PC-3.50CNT	99.9481%	0.007591 s
PC-4.00CNT	99.9041%	0.007694 s
PC-4.50CNT	99.9102%	0.011504 s
PC-5.00CNT	99.9448%	0.007437 s
PC-10.0CNT	99.9373%	0.007264 s
PC-15.0CNT	99.9326%	0.007259 s
PC-20.0CNT	99.8815%	0.007183 s
Mean	99.7997%	0.008440 s
